# Spinal pain in adolescents: prevalence, incidence, and course: a school-based two-year prospective cohort study in 1,300 Danes aged 11–13

**DOI:** 10.1186/1471-2474-15-187

**Published:** 2014-05-29

**Authors:** Ellen Aartun, Jan Hartvigsen, Niels Wedderkopp, Lise Hestbaek

**Affiliations:** 1 Department of Sports Science and Clinical Biomechanics, University of Southern Denmark, Odense, Denmark; 2 Nordic Institute of Chiropractic and Clinical Biomechanics, Odense, Denmark; 3 Institute of Regional Health Services Research, Sygehus Lillebælt, University of Southern Denmark, Middelfart, Denmark

**Keywords:** Prevalence, Incidence, Frequency, Intensity, Course, Spinal pain, Adolescence

## Abstract

**Background:**

The severity and course of spinal pain is poorly understood in adolescents. The study aimed to determine the prevalence and two-year incidence, as well as the course, frequency, and intensity of pain in the neck, mid back, and low back (spinal pain).

**Methods:**

This study was a school-based prospective cohort study. All 5th and 6th grade students (11–13 years) at 14 schools in the Region of Southern Denmark were invited to participate (N = 1,348). Data were collected in 2010 and again two years later, using an e-survey completed during school time.

**Results:**

The lifetime prevalence of spinal pain was 86% and 89% at baseline and follow-up, respectively. A group of 13.6% (95% CI: 11.8, 15.6) at baseline and 19.5% (95% CI: 17.1, 22.0) at follow-up reported that they had pain frequently. The frequency of pain was strongly associated with the intensity of pain, i.e., the majority of the participants reported their pain as relatively infrequent and of low intensity, whereas the participants with frequent pain also experienced pain of higher intensity. The two-year incidence of spinal pain varied between 40% and 60% across the physical locations. Progression of pain from one to more locations and from infrequent to more frequent was common over the two-year period.

**Conclusions:**

Spinal pain is common at the age of 11–15 years, but some have more pain than others. The pain is likely to progress, i.e., to more locations, higher frequency, and higher pain intensity over a two-year period.

## Background

It is now widely acknowledged that neck pain (NP), mid back pain (MBP), and low back pain (LBP) (spinal pain) start early in life and that the lifetime prevalence increases rapidly during adolescence to reach adult levels at the age of 18 [1,2]. In adults, LBP is now the leading cause of years lived with disability on a global level [3] and the societal burden due to disability pensions and treatment costs for this disorder are high and increasing [4]. Even amongst adolescents, consequences of pain are common, e.g., 8% of all 13 year olds and 34% of all 15-year olds seek health care for spinal pain in Denmark [5], and among those reporting recurrent LBP, 31% have refrained from participating in sport and physical activity and 26% have been absent from school [6], indicating that spinal pain in adolescence is not a negligible problem. In addition, spinal pain in adolescence is strongly associated with spinal pain and generalised pain in adulthood [7,8] so, the young population is a group of special interest when exploring the incidence, progression, and severity of spinal pain.

The incidence of NP, MBP, and LBP reported in adolescents is based on various definitions of pain, i.e., weekly pain in the past 3 or 6 months [9-12], frequent pain [13], quite bad pain [14], and pain in the past week [15]. Incidence is however defined as new cases of a disease during a specified time period and prior studies have rarely been based on a group that has never had pain. In addition, the severity of spinal pain in adolescents has previously been assessed by describing the consequences of the pain and not directly by describing the pain characteristics such as frequency and intensity [16]. In addition, the aspect of multiple pain sites and the extent to which the pain dissipates or changes location in the spine over time has not been addressed in this population, which is of interest because multiple pain sites are associated with high disability in adolescents [17], and in adults, they are a predictor of work disability [18]. Consequently, there has been a call for more longitudinal studies [19], but to our knowledge, there are no population-based longitudinal studies that describe pain characteristics and changes in spinal pain in adolescence.

Therefore, the overall aims of this longitudinal cohort study were to determine the prevalence, severity and course of spinal pain in Danish adolescents. The specific objectives were to:

a) Determine the lifetime, one-week, and point prevalence of NP, MBP, and LBP (separately and combined) at age 11–13 and age 13–15,

b) Determine the frequency and intensity of NP, MBP, and LBP and explore the associations between the two,

c) Estimate the two-year incidence of NP, MBP, and LBP, and

d) Describe the changes in pain location and the changes in frequency of spinal pain from baseline to follow-up.

## Methods

### Participants

This study was nested within the SPACE study. SPACE was a school-based cluster-randomised controlled trial involving 14 schools in the Region of Southern Denmark [20]. The main aim of SPACE was to investigate how physical environment combined with organisational initiatives could promote physical activity in adolescents aged 11–13 years. All 1,348 5th and 6th grade students (aged 11–13 years) at the 14 schools were invited to participate. There were no exclusion criteria. For a comprehensive description, see the SPACE protocol [20]. There were no differences between the control group and the intervention group at baseline or follow-up with regard to adiposity, physical fitness or musculoskeletal strength [21]. Therefore, it did not seem likely that the intervention had an impact on the presence of spinal pain and so, we decided to use the pooled cohort rather than the control group only.

Prior to the study, the parents of the involved students received a letter including information about the project. Participation did not require parental consent, but the parents were informed that they could initially, or at any time later, withdraw their child’s participation. This form of passive consent was reviewed by the Regional Committee for Health Research Ethics together with the rest of the project protocol. The conclusion was that the project was acceptable according to Danish legislation and did not require formal approval because all tests were non-invasive and there were no physical interventions involved [22]. Approval from the Danish Data Protection Agency was obtained (#2010-41-5147).

### Data collection

The participants completed an electronic questionnaire (e-survey) during school time, observed by a teacher who also ensured that there were no interactions between participants. For a detailed description of the e-survey, see the SPACE protocol [20]. Baseline data were collected from April to June 2010 and two-year follow-up data from April to June 2012.

The e-survey contained parts of the Young Spine Questionnaire [23], which included identical questions for the three spinal regions separately. Using the neck questions as an example, the first question was: "Have you ever had pain in your neck?" ("often"/"sometimes"/ "once or twice"/"never"). If so, the next questions were: "Have you had neck pain in the last week?" ("yes"/"no") and "Do you have neck pain today?" ("yes"/"no"). Finally, they noted the worst pain ever in the neck using the revised version of the Faces Pain Scale (FPS-R) [24]. This scale is based on six faces with expressions illustrating progressively worse pain. The questions were repeated for the mid back and low back. A diagram with the three spinal areas clearly shaded and labelled was shown alongside the questions. The e-survey was designed in such a way that there was a jump to the next spinal region if the participant answered "never" to a question because any subsequent questions about pain last week or today, and pain intensity then became irrelevant for that particular spinal region. Immediately following the questions, an open field for comments was provided. The questionnaire was developed for 9-11-year olds and has shown satisfactory results for feasibility, content validity, and item agreement between questionnaire scores and interview findings [23].

### Variables

• Lifetime, one week, and point prevalence of NP, MBP, and LBP were defined as a positive response to the first, second and third questions respectively for each spinal region. If the student answered "never" to the lifetime question, it was assumed that the answers to the one week and point prevalence questions were "no". Spinal pain was defined as pain in any of the three locations.

• Frequency of NP, MBP, and LBP were "often", "sometimes", "once or twice", or "never". Frequency of spinal pain was defined as "often", "sometimes", "once or twice", or "never" in any of the three locations. If the frequency of pain differed in the three locations, the location with the highest frequency was used. A missing value in any region resulted in a missing value for this variable.

• Pain intensity was based on the FPS-R with six faces scaled from 1 to 6 where 1 was labelled "no pain" and 6 as "very much pain" in the questionnaire. Then, the pain intensity was rescaled into a 0–10 scale (1 → 0, 2 = 2, 3 → 4, 4 → 6, 5 → 8, and 6 → 10).

• Incidence cases were defined as those who reported "never" having had pain at baseline but reporting pain at follow-up. Incidence proportions were reported separately for each spinal location.

• Pain locations were defined as none, NP only, MBP only, LBP only, NP + MBP, NP + LBP, MBP + LBP, and NP + MBP + LBP based on answers to the lifetime question. A missing value in any region resulted in a missing value for all the pain combinations that included this region.

### Descriptive statistics

The prevalence, frequency, two-year incidence, and changes in pain locations and frequency of spinal pain from baseline to follow-up were reported using percentages and 95% confidence intervals (CI). The pain intensity was reported using the mean with 95% CI. Cross-sectional descriptions were reported by gender, but due to smaller cell sizes, the longitudinal descriptions were presented for the combined cohort only. The associations between frequency and pain intensity were illustrated graphically by histograms with 95% CI bars.

Post-hoc analysis: There was a technical problem at one school where some of the students were unable to respond "never" at the LBP question at baseline. The lifetime, one week and point prevalence of LBP were therefore calculated also with this school excluded in order to investigate how much this technical problem had affected the prevalence of LBP. All statistics were calculated using STATA version 11.2.

## Results

The participation rate was 95.8% (n = 1,291) at baseline and 82.4% (n = 1,064) at follow-up. Seventy-seven percent (n = 1,042) filled in both questionnaires. The participation rate for the follow-up was lower, primarily because a number of students had changed school (n = 156). Nineteen students refused to participate at baseline, and a further 14 refused to participate at follow-up. There were 51.6% (n = 666) boys at baseline and 53.1% (n = 565) at follow-up. The mean age was 12.6 years (SD = 0.63) at baseline. There were some statistically significant differences in baseline characteristics of those who completed both questionnaires and those who just completed the baseline questionnaire. Among the drop-outs, there was a predominance of girls and participants who had a history of NP and LBP, and participants who had pain at more pain sites, but who did not have higher frequency or intensity of pain.

### Prevalence

At baseline, the lifetime prevalence of spinal pain was 86% (95% CI: 84.0, 87.8), the one-week prevalence was 35.9% (95% CI: 33.3, 38.6) and the point prevalence was 16.9% (95% CI: 14.9, 19.0). At follow-up, the lifetime, one-week and point prevalence of spinal pain was 88.8% (95% CI: 86.9, 90.7), 48.5% (95% CI: 45.4, 51.5) and 22.9% (95% CI: 20.4, 25.5) respectively. NP was consistently the most common spinal pain site followed by MBP, and lastly LBP (Table 1). The lifetime, one week and point prevalence at baseline and follow-up are for all locations presented by gender in Table 1.

**Table 1:** Lifetime, one week, and point prevalence of neck, mid back, and low back pain at age 11–13 and two years later

		**11-13 yr. (n = 1291)**						**13-15 yr. (n = 1064)**					
		**Girls (n = 625)**			**Boys (n = 666)**			**Girls (n = 499)**			**Boys (n = 565)**		
		**n (mis)**	**%**	**95% CI**	**n (mis)**	**%**	**95% CI**	**n (mis)**	**%**	**95% CI**	**n (mis)**	**%**	**95% CI**
**Lifetime prevalence**	NP	491	78.6	75.3, 81.2	490	73.6	70.2, 76.9	408	81.8	78.4, 85.2	410	72.6	68.9, 76.2
	MBP	382	61.1	57.3, 64.9	387 (1)	58.2	54.4, 61.9	339	67.9	63.8, 72.0	336 (1)	59.6	55.5, 63.6
	LBP	302 (2)	48.5	44.6, 52.4	271 (1)	40.8	37.0, 44.5	293 (1)	58.8	54.5, 63.2	258 (1)	45.7	41.6, 49.9
**One week prevalence**	NP	149	23.8	20.5, 27.2	150	22.5	19.3, 25.7	164	32.9	28.7, 37.0	151 (2)	26.8	23.2, 30.5
	MBP	105 (1)	16.8	13.9, 19.8	132 (1)	19.8	16.8, 22.9	144 (3)	29.0	25.0, 33.0	136 (3)	24.2	20.7, 27.7
	LBP	82 (2)	13.2	10.5, 15.8	66 (1)	9.9	7.7, 12.2	108 (2)	21.7	18.1, 25.4	103 (5)	18.4	15.2, 21.6
**Point prevalence**	NP	68 (1)	10.9	8.5, 13.3	70 (2)	10.5	8.2, 12.9	81 (3)	16.3	13.1, 19.6	66 (4)	11.8	9.1, 14.4
	MBP	42	6.7	4.8, 8.7	62 (1)	9.3	7.1, 11.5	47 (2)	9.5	6.9, 12.0	52 (3)	9.3	6.9, 11.6
	LBP	31 (2)	5.0	3.3, 6.7	28 (1)	4.2	2.7, 5.7	40 (3)	8.1	5.7, 10.5	33 (6)	5.9	3.9, 7.9

CI = Confidence intervals; LBP = Low back pain; MBP = Mid back pain; Mis = Missing values; NP = Neck pain.

### Frequency, pain intensity, and the association between them

NP, MBP, and LBP were mostly experienced only "once or twice" at both time-points (Table 2). A group of 13.6% (95% CI: 11.8, 15.6) of the participants reported that they often had spinal pain at baseline and this group increased to 19.5% (95% CI: 17.1, 22.0) at follow-up. Detailed results for the frequency of NP, MBP, and LBP are presented by gender in Table 2.

**Table 2:** Frequency of neck, mid back, and low back pain at age 11–13 and two years later

		**11-13 yr. (n = 1291)**						**13-15 yr. (n = 1064)**					
		**Girls (n = 625)**			**Boys (n = 666)**			**Girls (n = 499)**			**Boys (n = 565)**		
**Location**	**Frequency**	**n**	**%**	**95% CI**	**n**	**%**	**95% CI**	**n**	**%**	**95% CI**	**n**	**%**	**95% CI**
**NP**	Never	134	21.4	18.2, 24.7	176	26.4	23.1, 29.8	91	18.2	14.9, 21.6	155	27.4	23.8, 31.1
	Once/twice	264	42.2	38.4, 46.1	252	37.8	34.2, 41.5	177	35.5	31.3, 39.7	194	34.3	30.4, 38.3
	Sometimes	179	28.6	25.1, 32.2	196	29.4	26.0, 32.9	183	36.7	32.4, 40.9	168	29.7	26.0, 33.5
	Often	48	7.7	5.6, 9.8	42	6.3	4.5, 8.2	48	9.6	7.0, 12.2	48	8.5	6.2, 10.8
	Missing values	0			0			0			0		
**MBP**	Never	243	38.9	35.1, 42.7	278	41.8	38.1, 45.5	160	32.1	38.0, 36.2	228	40.4	36.4, 44.5
	Once/twice	234	37.4	33.6, 41.2	229	34.4	30.8, 38.1	180	36.1	31.9, 40.3	183	32.5	28.6, 36.3
	Sometimes	112	17.9	14.9, 20.9	115	17.3	14.4, 20.2	117	23.5	19.7, 27.2	108	19.2	15.9, 22.4
	Often	36	5.8	3.9, 7.6	43	6.5	4.6, 8.3	42	8.4	6.0, 10.9	45	8.0	5.7, 10.2
	Missing values	0			1			0			1		
**LBP**	Never	321	51.5	47.6, 55.4	394	59.3	55.5, 63.0	205	41.2	36.8, 45.5	306	54.3	50.1, 58.4
	Once/twice	191	30.7	27.0, 34.3	181	27.2	23.8, 30.6	141	28.3	24.4, 32.3	143	25.4	21.8, 28.9
	Sometimes	73	11.7	9.2, 14.2	64	19.6	7.4, 11.9	92	18.5	15.1, 21.9	86	15.3	12.3, 18.2
	Often	38	6.1	4.2, 8.0	26	3.9	2.4, 5.4	60	12.1	9.2, 14.9	29	5.1	3.3, 7.0
	Missing values	2			1			1			1		

CI = Confidence intervals; LBP = Low back pain; MBP = Mid back pain; NP = Neck pain.

**Figure 1:**
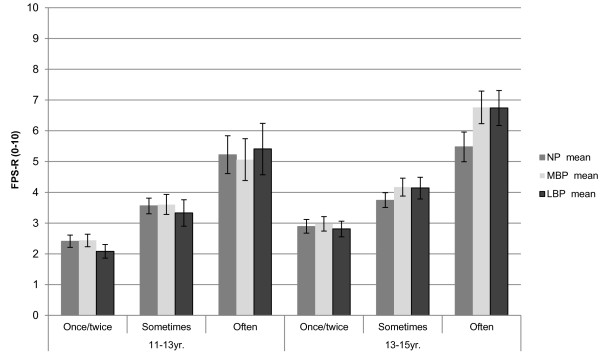
**The association between frequency and pain intensity at age 11–13 and two years later.** FPS-R = Faces pain scale – revised; LBP = Low back pain; MBP = Mid back pain; NP = Neck pain. Error bars show 95% confidence intervals.

The mean pain intensity was relatively low at baseline for all three spinal regions: 3.1 (95% CI: 2.9, 3.3) for NP, 3.1 (95% CI: 2.9, 3.2) for MBP and 2.7 (95% CI: 2.5, 3.0) for LBP. At follow-up, the mean pain intensity was higher in all locations. See Table 3 for gender-specific details.The mean pain intensity was lowest for students reporting pain "once or twice" with a statistically significant progressive increase across the "sometimes" and "often" groups. Thus the largest group had pain that was both of low frequency and low intensity, but a smaller group reported pain of both high frequency and intensity. This pattern was seen for all spinal regions and at both time-points (Figure 1).

**Table 3:** Intensity of neck, mid back, and low back pain (FPS-R 0–10 scale) at age 11–13 and two years later

	**11-13 yr. (n = 1291)**						**13-15 yr. (n = 1064)**					
	**Girls (n = 625)**			**Boys (n = 666)**			**Girls (n = 499)**			**Boys (n = 565)**		
**Location**	**n (mis)**	**Mean intensity**	**95% CI**	**n (mis)**	**Mean intensity**	**95% CI**	**n (mis)**	**Mean intensity**	**95% CI**	**n (mis)**	**Mean intensity**	**95% CI**
NP	488 (3)	3.3	3.0, 3.5	489 (1)	3.0	2.7, 3.2	405 (3)	3.8	3.6, 4.0	408 (2)	3.4	3.4, 3.6
MBP	378 (4)	3.3	3.1, 3.6	387 (1)	2.8	2.5, 3.0	338 (1)	3.9	3.6, 4.2	332 (5)	3.8	3.5, 4.1
LBP	300 (4)	2.9	2.6, 3.2	271 (1)	2.5	2.2, 2.8	289 (5)	4.1	3.8, 4.4	252 (7)	3.6	3.3, 3.9

CI = Confidence intervals; FPS-R = Faces Pain Scale – Revised; LBP = Low back pain; MBP = Mid back pain; Mis = Missing values; NP = Neck pain.

### Two-year incidence

The two-year incidence of NP (no NP at baseline followed by NP at follow-up) was 60.1% (95% CI: 54.1, 66.0), for MBP 49.8% (95% CI: 45.0, 54.5), and for LBP 42.1% (95% CI: 38.2, 46.1).

### Changes in pain locations

Both at baseline and follow-up, pain in all three locations combined was most common followed by the combination of NP and MBP (Table 4). We observed that changes in pain location were common over the two-year period (Table 4). We also observed that regardless of pain location at baseline, they were most likely to report pain in all three locations at follow-up. An exception to this was those with MBP only, who were more likely to have the combination of NP and MBP at follow-up (Table 4). In total, 378 (51.8%) participants had pain in more locations at follow-up than at baseline, whereas 262 (29.8%) had pain in fewer locations.

**Table 4:** Changes in location of spinal pain from age 11–13 to 13–15

	**Pain locations 13–15 yr.**																
	**None (n = 119)**		**NP only (n = 143)**		**MBP only (n = 53)**		**LBP only (n = 43)**		**NP + MBP (n = 184)**		**NP + LBP (n = 78)**		**MBP + LBP (n = 26)**		**NP + MBP + LBP (n = 392)**		**Total**
**Pain locations 11–13 yr.**	**%**	**95% CI**	**%**	**95% CI**	**%**	**95% CI**	**%**	**95% CI**	**%**	**95% CI**	**%**	**95% CI**	**%**	**95% CI**	**%**	**95% CI**	**%**
None (n = 158)	32.3	25.1, 40.2	17.7	12.1, 24.6	5.7	2.6, 10.5	7.6	4.0, 12.9	13.9	8.9, 20.3	4.4	1.8, 8.9	3.2	1.0, 7.2	15.2	10.0,21.8	100
NP only (n = 169)	13.0	8.3, 19.0	20.1	14.4, 27.0	4.7	2.1, 9.1	3.0	1.0, 6.8	16.9	11.3, 23.0	10.7	6.4, 16.3	3.0	1.0, 6.8	29.0	22.3, 36.5	100
MBP only (n = 54)	7.4	2.1, 17.9	14.8	6.6, 27.2	13.0	5.4, 24.9	5.6	1.2, 15.4	29.6	18.0, 43.6	3.7	0.5, 12.7	5.6	1.2, 15.4	20.4	10.6, 33.5	100
LBP only (n = 23)	4.4	0.1, 21.9	13.0	2.8, 33.6	0.0	0.0, 14.8	13.0	2.8, 33.6	13.0	2.8, 33.6	8.7	1.1, 28.0	4.4	0.1, 21.9	43.5	23.2, 65.5	100
NP + MBP (n = 224)	9.4	5.9, 14.0	12.5	8.5, 17.6	6.7	3.8, 10.8	3.6	1.6, 6.9	21.9	16.6, 27.9	6.7	3.8, 10.8	1.3	0.3, 3.9	38.0	31.6, 44.7	100
NP + LBP (n = 76)	6.6	2.2, 14.7	14.5	7.5, 24.4	4.0	0.8, 11.1	5.3	1.5, 12.9	17.1	9.4, 27.5	10.5	4.7, 19.7	4.0	0.8, 11.1	38.2	27.2, 50.0	100
MBP + LBP (n = 26)	11.5	2.4, 30.2	7.7	0.9, 25.1	7.7	0.9, 25.1	0.0	0.0, 13.2	26.9	11.6, 47.8	11.5	2.4, 30.2	0.0	0.0, 13.2	34.6	17.2, 55.7	100
NP + MBP + LBP (n = 308)	3.9	2.0, 6.7	9.4	6.4, 13.2	2.9	1.3, 5.5	2.6	1.1, 5.1	14.9	11.1, 19.4	7.5	4.8, 11.0	2.0	0.7, 4.2	56.8	51.1, 62.4	100

CI = Confidence intervals; LBP = Low back pain; MBP = Mid back pain; NP = Neck pain. There are 4 missing values in the table.

Proportions of adolescents with different combinations of baseline categories (rows) and follow-up categories (columns).

### Changes in frequency of spinal pain

It appears that the spinal pain progressed from "never" to "once or twice" and further to "sometimes" and "often". Only a very small proportion of participants shifted from "never" to "often" (1.9%) (Table 5) and half of those reporting spinal pain "often" at baseline continued to have it at follow-up (Table 5). Paradoxically, a small proportion (7.7%) of those who reported pain at baseline did not do so at follow-up. We ascribe this to poor recall, and it is worth noting that this proportion was lower for those who reported spinal pain "often" (3.9%) than those who reported spinal pain "once or twice" (9.3%) at baseline (Table 5).

**Table 5:** Changes in frequency of spinal pain from age 11–13 to 13–15

	**Frequency of spinal pain 13–15 yr.**												
**Frequency of spinal pain 11–13 yr.**	**Never (n = 119)**			**Once or twice (n = 345)**			**Sometimes (n = 372)**			**Often (n = 202)**			**Total**
	**n**	**%**	**95% CI**	**n**	**%**	**95% CI**	**n**	**%**	**95% CI**	**n**	**%**	**95% CI**	**%**
Never (n = 158)	51	32.3	25.1, 40.2	60	38.0	30.4, 46.0	44	27.9	21.0, 35.5	3	1.9	0.4, 5.4	100
Once or twice (n = 407)	38	9.3	6.7, 12.6	185	45.5	40.5, 50.4	138	33.9	29.3, 38.7	46	11.3	8.4, 14.8	100
Sometimes (n = 346)	25	7.2	4.7, 10.5	86	24.9	20.4, 29.8	145	41.9	36.7, 47.3	90	26.0	21.5, 31.0	100
Often (n = 127)	5	3.9	1.3, 8.9	14	11.0	6.2, 17.8	45	35.4	27.2, 44.4	63	49.6	40.6, 58.6	100

CI = Confidence intervals. There are 4 missing values in the table.

Proportions of adolescents with different combinations of baseline categories (rows) and follow-up categories (columns).

### Post-hoc analysis

When the school with the technical failure was excluded from analysis, the lifetime prevalence of LBP at baseline was two percentage points lower than reported in Table 1, but there were no differences in the one-week and point prevalence.

## Discussion

Pain in the spine affected almost nine out of ten 11-15-year olds and among those who did not report spinal pain at age 11–13, around half had experienced pain two years later. For the majority, the pain appears to be relatively mild, i.e., mostly reported as "once or twice" and of low intensity. However, 14-20% reported more frequent pain which was also of higher intensity. In addition, localised spine pain in early adolescence appears to spread to involve other areas of the spine over time.

This study reports higher prevalence and incidence of spinal pain than previous studies. A systematic review has shown that the lifetime prevalence of NP in adolescents ranged from 3% to 8%, MBP from 9.5% to 72%, and LBP from 7% to 72% [1]. Earlier reporting of incidence has been based on varying definitions including weekly pain in the past 3 or 6 months [9-12], frequent pain [13], quite bad pain [14], and pain in the past week [15]. Nissinen et al. reported a one-year incidence of LBP, based on ‘LBP ever’, in 18% of adolescents from age 13 to 14 [25], which appears comparable to our result of 42% for a two-year incidence. However, because of the large variation in pain reporting in the past, comparison between estimates should be made with caution [1]. A reasonable explanation for the high lifetime prevalence and incidence in this study is related to the response options of the first question ("Have you ever had neck pain?") which were "often"/"sometimes"/"once or twice"/"never". We assume some of those answering "once or twice" might have answered "no" if the only options were "yes" or "no". This assumption is based on an issue detected during the development of the questionnaire. In the first version, there was not a "once or twice" response option. During interviews, it became obvious that many children did not know whether to answer "never" or "sometimes" if the pain was experienced only once or twice, as they did not consider this frequency enough to be categorised as "sometimes". Therefore, the option "once or twice" was included in the questionnaire [23]. The theory of the "once or twice" response option as an explanation of the high lifetime prevalence could be supported by another observation. If the variable of lifetime prevalence was dichotomised differently, i.e., if "once or twice" was included as "no", the results were more similar to previous reported lifetime prevalence. Although it makes comparisons with previous studies difficult, we recommend this approach in the future as it might result in more precise estimates. Also, some considerations need to be taken into account when presenting incidence rates. We observed that some students at follow-up reported that they had "never" had pain, despite a report of pain at baseline. Therefore, it is likely that some of those who reported never having had pain at baseline could have had an experience of pain previously. Since incidence is defined as new cases of a disease over a specified time period, we are sceptical of the use of this term.

The literature about frequency, pain intensity and the association between them is currently limited, but supports the findings from this study. Investigating back pain frequency, Brattberg et al. reported that 22% of the boys in their study and 47% of the girls aged 13 years had back pain often [13]. A similar association between frequency and intensity was reported in another study for NP [10], but no studies of such an association were found in relation to MBP and LBP.

The report of pain in multiple locations was more common than reporting pain in one location only. We know that multiple pain sites are associated with disability in adolescents [17], but it is unknown if there is a difference in disability between those who experience pain simultaneously at the three locations or those with pain that shifts from one location to another. The association between disability and concurrent/non-concurrent pain at multiple sites, and the influence of other pain characteristics such as frequency and intensity should be investigated in future research.

The strengths of this study were the school-based population and the longitudinal design. A follow-up period of two years is relatively short from a life course perspective, but enough to be able to observe an increase in all measured parameters and a noticeable change in pain location and frequency of spinal pain. Another strength was that the questionnaire used was developed for the target population [23] and answered by the participants without parental or peer influence. We know that this age group is not very discriminative when reporting pain, e.g., scratches can be reported as back pain if they are located on the back, so comparison of prevalence with the adult population should be done with caution [23]. This is one of the reasons why we included other descriptive variables like frequency and pain intensity in the study; we wanted other variables to determine the severity of the pain.

The technical problem at one of the schools slightly inflated the lifetime prevalence of LBP at baseline, but did not influence the conclusions that can be drawn from this study. Finally, biological age might be a better predictor than chronological age as used in this study, but unfortunately we do not have data to illustrate this. We recommend that pubertal stage is considered in future studies.

## Conclusions

Neck, mid back, and low back pain are common at the age of 11–15 years. For the majority of the participants, the pain seems to be mild in nature, relatively infrequent and of low intensity. A group of 14-20% was more severely affected with frequent pain which was also more intense. The two-year course showed a progressive development in pain, and that pain was likely to spread to more locations over a two-year period, regardless of initial pain location.

## Abbreviations

CI: Confidence interval; FPS-R: Faces pain scale – revised; LBP: Low back pain; MBP: Mid back pain; NP: Neck pain.

## Competing interests

The authors declare that they have no competing interests.

## Authors’ contributions

EA: Conception and design, analysis, drafting the manuscript. JH: Conception and design, analysis, revising the manuscript. NW: Conception and design, revising the manuscript. LH: Conception and design, data collection, analysis, revising the manuscript. All authors have read and approved the manuscript.

## Pre-publication history

The pre-publication history for this paper can be accessed here:

http://www.biomedcentral.com/1471-2474/15/187/prepub
